# Early Adverse Events between mRNA and Adenovirus-Vectored COVID-19 Vaccines in Healthcare Workers

**DOI:** 10.3390/vaccines9080931

**Published:** 2021-08-21

**Authors:** Yu-Mi Wi, Si-Ho Kim, Kyong-Ran Peck

**Affiliations:** 1 Division of Infectious Diseases, Samsung Changwon Hospital, Sungkyunkwan University, Changwon 51353, Korea; yrhg95@naver.com (Y.-M.W.); wychhazel@naver.com (S.-H.K.); 2 Division of Infectious Diseases, Samsung Medical Center, Sungkyunkwan University School of Medicine, Seoul 06351, Korea

**Keywords:** coronavirus disease 2019, vaccine, adverse event, BNT162b2 mRNA vaccine, AZD1222 vaccines

## Abstract

Mass vaccination campaigns are important to control the COVID-19 pandemic, however, adverse events (AEs) contribute to vaccine hesitancy. To investigate and compare early AEs between the BNT162b2 mRNA and AZD1222 adenovirus-vectored vaccines, recipients completed daily surveys about local and systemic reactions for 7 days after each dose, respectively. A total of 80 and 1440 healthcare workers received two doses of BNT162b2 and a first dose of AZD1222 vaccines. Any AEs were reported by 52.5% of recipients after the first dose of BNT162b2, by 76.2% after the second dose of BNT162b2, and by 90.9% after the first dose of AZD1222 (*p* < 0.001). Younger vaccinees had more AEs after the second dose of BNT162b2 and first dose of AZD1222. Sex based differences were only observed in the AZD1222 recipient group. No incidence of anaphylaxis or neurologic AEs were observed. In conclusion, early AEs were mostly mild to moderate in severity and generally transient in both BNT162b2 and AZD1222 groups. Sufficient explanation of the expected AEs of the vaccine would be helpful for wider vaccination.

## 1. Introduction

Mass vaccination campaigns are important to control the COVID-19 pandemic. As of 21 May 2021, four COVID-19 vaccines, the AZD1222 vaccine from Oxford/AstraZeneca, the BNT162b2 vaccine from Biontech and Pfizer, the Ad26.COV2.S vaccine from Johnson & Johnson (Janssen) and the mRNA-1273 vaccine from Moderna, were approved in Korea [1]. The AZD1222 vaccination program began for long-term care facility residents on 26 February 2021, while the BNT162b2 vaccination program was implemented for healthcare workers who encountered COVID-19 patients on 27 February 2021. As of 15 May 2021, a total of 7.2% of Koreans have received at least one dose of BNT162b2 or AZD1222 [1]. Because of the unparalleled speed of COVID-19 vaccine development with diverse technologies, the public has been concerned about the safety of COVID-19 vaccines [2,3]. Although the incidence rates of AEs of the BNT162b2 and the AZD1222 vaccines were different in previous clinical studies, the method of monitoring AEs might have resulted in the differences [4,5]. Therefore, continued monitoring of the AEs of COVID-19 vaccines in real-world settings may provide additional information for health care practitioners and the public to prevent fear of side effects and vaccine hesitancy. In this study, the AEs after each dose of the BNT162b2 mRNA and the first dose of the AZD1222 vaccines were surveyed to evaluate and compared using a self-reported form in a group of healthcare workers (HCWs).

## 2. Materials and Methods

Samsung Changwon Hospital is a university-affiliated hospital, whose eight negative-pressure isolation rooms were prepared for severe COVID-19 patients. According to Korean COVID-19 vaccination plans, the initial priorities were healthcare workers (HCWs) who were dedicated to COVID-19 patients, and residents and workers of long-term care facilities [6]. The BNT162b2 vaccine was assigned to 80 high-risk HCWs in direct contact with COVID-19 patients and AZD1222 vaccines were for the rest of the HCWs involved in general patient care. Between 3 March 2020 and 26 March 2020, HCWs were scheduled to receive either the AZD1222 or the BNT162b2 vaccine. Individuals with a history of severe allergic reactions to the components of the COVID-19 vaccine, those who were pregnant, and those who refused to be vaccinated for personal reasons were excluded from immunization candidates. Only one nurse tested positive for SARS-CoV-2 infection before vaccination. No one has tested positive for SARS-CoV-2 infection after getting vaccinated until 12 August 2021. After obtaining consent, HCWs were vaccinated. To screen and manage adverse events, the center for infection prevention and control of our hospital requested that all vaccine recipients report their symptoms daily 1 to 7 days after vaccination. Symptoms were recorded in a self-reporting form. The reported AEs were classified as previously reported [7]. Severe events were defined as stage 3 or higher symptoms, altered mental status/seizure/paralysis, and suspicious for anaphylaxis. We compared the frequencies and characteristics of AEs between vaccines. Discrete data were presented as frequencies and percentages, and continuous variables were summarized as the mean ± standard deviation or the median and interquartile range after the normality of data were tested using the Shapiro–Wilk normality test. Student’s t-test or Mann–Whitney tests were used to compare the continuous variables of the two groups. Categorical variables were compared using the chi-square test or Fisher’s exact test. All *p* values were two-tailed, and *p* values < 0.05 were considered statistically significant. All analyses were conducted with SPSS Statistics 23.0 for Windows (IBM, 2015, Chicago, IL, USA).

## 3. Results

During the vaccination program, 80 and 1440 HCWs received two doses of the BNT162b2 and one dose of the AZD1222 vaccines, respectively. The BNT162b2 group had significantly more male HCWs than the AZD1222 group; however, there was no significant difference in occupation type and age (Table 1).

**Figure 1:**
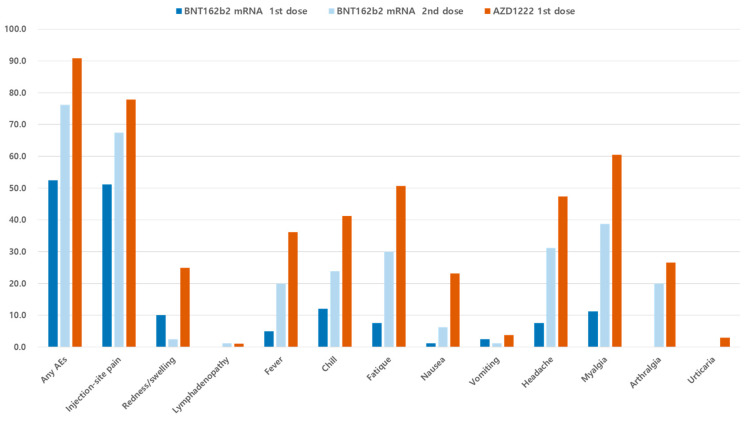
Incidence of reported adverse events in the first and the second vaccine recipients. AEs, adverse events.

A total of 80 and 1431 HCWs in each vaccine group reported symptoms at least once in the seven days post vaccination. Figure 1 and Table 1 display the AEs reported after the first and second doses of BNT162b2 and one dose of AZD1222 vaccines, respectively. When comparing AEs after the first and second doses of BNT162b2, reported AEs were more common in recipients of the second dose than in those recipients of the first dose (76.2% vs. 52.5%, *p* = 0.003). Additionally, recipients of the second dose of BNT162b2 were seven times more likely to need medication to alleviate AEs compared to those of the first vaccination (45.0% vs. 7.5%, *p* < 0.001). When comparing the second BNT162b2 dose with the one AZD1222 dose, AEs occurred more frequently after the AZD1222 dose than after the second BNT162b2 dose (90.9% vs. 76.2%, *p* < 0.001). Recipients of the AZD1222 vaccine were 1.6 times more likely to require medication to ameliorate their AEs compared to the second dose of BNT162b2 recipients (70.9% vs. 45.0%, *p* < 0.001). Typically, AEs occurred on the day following vaccination and lasted for one or two days. However, injection-site pain continued for an average of 3 days in the one dose of AZD1222 group.

Common AEs including injection-site pain (77.8% vs. 67.5% vs. 51.2%), myalgia (60.5% vs. 38.8% vs. 11.2%), headache (47.4% vs. 31.2% vs. 7.5%), fatigue (50.7% vs. 30.0% vs. 7.5%), chills (41.2% vs. 23.8% vs. 1.2%) and fever (36.1% vs. 20.0%, 5.0%) appeared to be the most frequent in the one dose of AZD1222 recipients, followed by the second dose of and the first dose of BNT162b2 vaccine. Urticaria was reported by 42 employees (2.9%) in the AZD1222 recipients alone. No anaphylaxis events or neurologic AEs were observed in any vaccine recipient.

In the second dose of the BNT162b2 vaccine, age related relationships were observed between patients with AEs (mean age: 33.57 years) and without AEs (mean age: 43.21 years) (*p* = 0.010). These age related differences were not observed with the first dose of BNT162b2 vaccine (Table 2). Younger vaccinees also had more AEs in the one dose of AZD1222 recipients (35.19 years vs. 42.34 years, *p* < 0.001). Sex related relationships were only observed in the AZD1222 recipient group (Table 2).

## 4. Discussion

Assessing vaccine hesitancy and ensuring that vaccination coverage is high enough to lead to herd immunity is essential to controlling the COVID-19 pandemic. The BNT162b2 vaccine is a lipid nanoparticle-formulated, nucleoside-modified RNA (mRNA) vaccine, whereas the AZD1222 vaccine is a replication-deficient chimpanzee adenovirus-vectored vaccine [8]. Different vaccine platforms have been expected to produce different AEs according to their platform [8]. Our study showed that a much higher incidence of mild-to-moderate AEs were reported after the AZD1222 vaccine than after each dose of the BNT162b2 vaccine. However, the overall safety of COVID-19 vaccines is reassuring, and no unexpected patterns of issues were identified. This finding is consistent with the reports of numerically higher incidences of AEs in those receiving the AZD1222 vaccine compared to those receiving the BNT162b2 vaccine [4,5,9,10]. At least one local symptom was reported in 88.0% and at least one systemic symptom was present in 86.0% of recipients between 18–55 years of age, after the first AZD1222 vaccination [4]. The incidence of an objectively measured fever within 7 days after the first vaccination with AZD1222 was 24% in vaccine recipients aged 18–55 years [4]. In contrast, a lower incidence of AEs was reported in BNT162b2 recipients [5,9]. Interestingly, more AEs were reported after the second dose than the first dose of the BNT162b2 vaccine: injection-site pain (75.2% vs. 70.0%), fatigue (53.9% vs. 30.9%), headache (46.7% vs. 25.9%), myalgia (44.0% vs. 19.4%), and fever (21.5% vs. 7.0%) [9]. AEs were less common in older adults than in younger adults in both AZD1222 and BNT162b2 recipients [4,5,9,10]. Consistent with prior data, our data showed that younger HCWs reported more AEs both in the second dose of the BNT162b2 and one dose of the AZD1222 vaccines.

Our study compared the incidence of AEs of both the BNT162b2 and the AZD1222 vaccines. Although this study is limited by the sample size, especially for the BNT162b2 vaccine, our findings are consistent with a previous study reporting more common AEs after the second dose than with the first dose of the BNT162b2 vaccine. Additionally, this study was similar to findings that showed that systemic adverse reactions occurred less frequently after both doses of the BNT162b2 vaccine than after the first dose of the AZD1222 vaccine [10,11]. In the previous mobile-based survey on the self-reported adverse reactions of the BNT162b2 vaccine, higher rates of AEs after the second dose were noted compared to the first dose (89.1% vs. 80.1%, *p* = 0.006) [11]. The frequencies of most AEs were significantly lower after the second dose of the BNT162b2 vaccine compared with the first dose of the AZD1222 vaccine. The large prospective community-based observational study of vaccines in the UK [10] compared AEs among recipients receiving one or two doses of the BNT162b2 vaccine or one dose of the AZD1222 vaccine. In the study, reported AEs were mostly of short duration and minor in severity [10]. In detail, systemic AEs were reported in 13.5% of recipients after the first dose of BNT162b2, in 22.0% after the second dose of BNT162b2 vaccine, and in 33.7% after the first dose of AZD1222 vaccine. Consistent with other studies, AEs occurred much more frequently in women than in men, in younger individuals, but at a much lower frequency than expected in other studies including ours [4,5,9]. These differences may be due to differences in the study population. The overall mean age of the UK study was higher than that of our study (61.5 years vs. 35.8 years). The low incidence of AEs in older age is a consistent result in all studies [4,5,9,10].

New reports of AEs have emerged as many additional people are vaccinated. The Committee of the European Medicines Agency has confirmed that that the AZD1222 vaccine may be associated with disseminated intravascular coagulation or cerebral venous sinus thrombosis [12]. Almost all reports of this serious condition involving low platelets with blood clots have occurred in adult women younger than 50 years of age [13]. On the other hand, anaphylaxis to the mRNA COVID-19 vaccine is currently estimated to occur in 2.5 to 11.1 cases per million doses [14]. Recently, myocarditis and pericarditis has been reported increasingly after mRNA COVID-19 vaccination in the United States [15]. Therefore, ongoing monitoring for rare and common adverse events after vaccination is important to evaluate the balance between risks and benefits for COVID-19 vaccines.

This study has several limitations. First, this study is limited to one institution with small sample size especially for the BNT162b2 vaccine, so the information in the data may not be representative or generalized. Second, self-reported symptoms are subjective rather than objective. However, most participants in this study were healthcare workers; therefore, self-reported data may be exceptionally reliable. Third, information on AEs that occurred more than 7 days after vaccination have not been collected and significant events may be missed. Finally, there were some unresponded data that could cause bias; however, the overall response rate exceeded more than 90%. The response rate of our study was higher than that of the prior clinical study. Despite these limitations, this study was important to evaluate and compare the AEs of HCWs vaccinated with the BNT162b2 or the AZD1222 vaccines in particular in HCWs, who are young.

## 5. Conclusions

In conclusion, the incidence of AEs was much higher among recipients of one dose of AZD1222 than in those with the first and the second dose of the BNT162b2 vaccine, although our sample size was limited in the case of the BNT162b2 vaccine. However, most AEs were mild to moderate in severity and resolved within 2 days. No unexpected patterns of AEs or other safety issues were identified during early monitoring. AEs contribute to vaccine hesitancy. Therefore, rapid collection and continuous monitoring of the safety of COVID-19 vaccines is critical to promoting vaccine policy and maintaining public confidence.

## Figures and Tables

**Table 1:** Adverse events following vaccination.

Characteristics	BNT162b2 (n = 80)		AZD1222 (n = 1431)	*p* Value *B 1st vs. B 2nd/ B 1st vs. A 1st/ B 2nd vs. A 1st
	After 1st Dose	After 2nd Dose	After 1st Dose	
Clinical characteristics				
Sex (male)	31 (38.8)		388 (27.1)	0.024
Age (mean ± SD)	35.83 ± 10.99		35.84 ± 11.13	0.993
Type of Occupation				0.078
Nurses and nursing assistants	44 (55)		743 (51.9)	
Doctors	18 (22.5)		206 (14.4)	
Other medical workers	7 (2.9)		235 (16.4)	
Non-medical staff and others	11 (4.3)		247 (17.3)	
All adverse events	42 (52.5)	61 (76.2)	1301 (90.9)	0.003/<0.001/<0.001
Adverse events requiring medication	6 (7.5)	36 (45.0)	1015 (70.9)	<0.001/<0.001/<0.001
Local reaction				
Pain at the injection site	41 (51.2)	54 (67.5)	1114 (77.8)	0.036/<0.001/0.032
Symptom onset	1 (1–1)	1 (1-1)	1 (1–1)	
Duration	2 (1–3)	2 (1–2)	3 (2–4)	
Redness/swelling at the injection site	8 (10.0)	2 (2.5)	352/1415 ^b^ (24.9)	0.050/0.002/<0.001
Symptom onset	1 (1–1)	1,2	2 (1–3)	
Duration	2 (2–2.75)	3,1	2 (1–3)	
Lymphadenopathy	0 (0.0)	1 (1.2)	14 (1.0)	>0.999/>0.999/0.811
Symptom onset		2	2 (1–3.5)	
Duration		2	1 (1–1)	
Systemic Events				
Fever	4 (5.0)	16 (20.0)	517 (36.1)	0.004/<0.001/<0.001
Mean ± SD	37.6 ^a^	38.1 ± 0.6	38.3 ± 0.6	0.211/0.754/>0.999
Symptom onset	1 (1–1)	1 (1–1)	1 (1–2)	
Duration	1 (1–1.75)	2 (1–2)	1 (1–2)	
Chills	1 (1.2)	19 (23.8)	589 (41.2)	<0.001/<0.001/0.002
Symptom onset	1	1 (1–2)	1 (1–1)	
Duration	1	1 (1–2)	1 (1–2)	
Fatigue	6 (7.5)	24 (30.0)	726 (50.7)	<0.001/<0.001/<0.001
Symptom onset	1 (1–1.25)	1 (1–2)	1 (1–2)	
Duration	2 (1.74–2.5)	2 (1–2)	2 (1–2)	
Nausea	1 (1.2)	5 (6.2)	327/1411 ^b^ (23.2)	0.210/<0.001/<0.001
Vomiting	2 (2.5)	1 (1.2)	53/1411 ^b^ (3.8)	0.999/0.765/0.250
Symptom onset	1,1	1.5 (1–2)	1 (1–2)	
Duration	2,3	1 (1–1)	1 (1–2)	
Headache	6 (7.5)	25 (31.2)	678 (47.4)	<0.001/<0.001/0.005
Symptom onset	1 (1–2.5)		1 (1–2)	
Duration	2 (1–2.25)		2 (1–2)	
Myalgia	9 (11.2)	31 (38.8)	866 (60.5)	<0.001/<0.001/<0.001
Symptom onset	1 (1–2)	2 (1–2)	1 (1–1)	
Duration	2 (1–2)	1 (1–2)	1 (1–2)	
Arthralgia	0 (0.0)	16 (20.0)	381 (26.6)	<0.001/<0.001/0.190
Symptom onset		1.5 (1–2)	1 (1–2)	
Duration		1 (1–2)	1 (1–2)	
Allergic reactions				
Urticaria	0 (0.0)	0 (0.0)	42 (2.9)	NA/0.166/0.166
Symptom onset			1 (1–2)	
Duration			1 (1–1)	

Data represent the number (%) of patients or the median value of days (interquartile range) unless otherwise specified. *B, BNT162b2; A, AZD1222. ^a^ Recorded fever in all four recipients was ≥37.6 °C. ^b^ There were 16 and 20 missing data or non-response instances for redness/swelling at the injection site and nausea/vomiting, respectively.

**Table 2:** Clinical characteristics of vaccine recipients with adverse events.

	Recipients with Adverse Event	Recipients without Adverse Event	*p*-Value
BNT162b2 Vaccine 1st	42 (52.5)	38 (47.5)	
Age	36.33 ± 9.46	35.26 ± 12.57	0.671
Sex			0.428
Male	18 (51.8)	13 (41.9)	
Female	24 (49.0)	25 (51.0)	
BNT162b2 Vaccine 2nd	61 (76.2)	19 (23.8)	
Age	33.57 ± 8.70	43.21 ± 14.20	0.010
Sex			0.462
Male	36 (59.0)	13 (68.4)	
Female	25 (41.0)	6 (31.6)	
AZT1222 1st	1301 (90.9)	130 (9.1)	
Age	35.19 ± 10.76	42.34 ± 12.64	<0.001
Sex			<0.001
Male	331 (85.3)	57 (14.7)	
Female	970 (93.0)	73 (7.0)	

## Data Availability

Data are available by emailing the corresponding author.
